# Evaluation of Bacterial Nanocellulose Membranes Loaded or Not with Nisin as a Complementary Treatment in Surgical Dehorning Wounds in Bovines

**DOI:** 10.3390/pharmaceutics13050688

**Published:** 2021-05-11

**Authors:** Fábio A. F. Custódio, Leonardo M. de Castro, Erick Unterkircher, Ana Carolina R. C. Porto, Iolanda S. Braga, Alessandre Hataka, Angela F. Jozala, Denise Grotto

**Affiliations:** 1 University of Sorocaba, Sorocaba 18023-000, Brazil; fabio.custodio@prof.uniso.br (F.A.F.C.); leonardo.castro@prof.uniso.br (L.M.d.C.); erick_unterkircher@hotmail.com (E.U.); carolina.porto@prof.uniso.br (A.C.R.C.P.); angela.jozala@prof.uniso.br (A.F.J.); 2 Department of Veterinary Clinical Sciences, School of Veterinary Medicine and Animal Science, São Paulo State University (UNESP), Botucatu 18610-307, Brazil; iolanda.braga@unesp.br (I.S.B.); hataka@fmvz.unesp.br (A.H.)

**Keywords:** bacterial nanocellulose, dehorning, dressing, wound healing, nisin, veterinary medicines

## Abstract

Treatments of postsurgical dehorning in cattle usually includes topical application of healing agents in wounds. The Bacterial Nanocellulose (BNC) may come to a complementary treatment for these wounds. Two new complementary treatments with BNC and BNC loaded with nisin were evaluated for wound healing in surgical dehorning in bovine. Hence, two groups of experiments were run, and 12 animals were used in each group. All animals, in right and left horns, received antisepsis treatment. For the first group, the left horn was the control, and on the right one the BNC was applied. For the second group, BNC was applied on the left horn (control) and on the right ones BNC+nisin was applied. In both experiments, wounds were evaluated macroscopically by photographic images and microscopically by histology. For macroscopic evaluations, a significant difference was observed over time, but only in the comparison within the same groups. Microscopic analyzes did not showed significant differences in any type of comparison. In conclusion, there was a clinical improvement in the wound healing response with the application of BNC. However, there was no significant difference between BNC compared to the nisin loaded in BNC. For the first time it was applied a BNC in surgical dehorning wounds in bovines and evaluated the efficacy of treatment in a real animal handling situation.

## 1. Introduction

The use of technologies which integrate the environment, natural resources and Animal Welfare (AW) practices is fundamental to achieve high productive efficiency levels in livestock production. Welfare practices include animal handling, i.e., the procedures to which animals are subjected to achieve the desired production efficiency. Some surgical procedures are performed to promote AW, for productive gain, and to ensure safety for handlers [1], since the procedures contribute to reducing the intensity of aggressive interactions among animals [2,3].

Surgical dehorning in adult animals is a procedure performed in cases of aggressiveness among animals, fractures, or abnormal conformation of the horns, despite being an invasive procedure [4]. Dehorning also contributes to reducing the frequency of lesions in udders, eyes, and flank region [5], besides promoting greater safety for handlers [1,6]. The surgical procedure consists of amputating the horn at its base after skin incision, followed by dermorrhaphy [7]. Some problems can arise during the procedure and in the postoperative period. The complications include dehiscence of surgical stitches due to the tension of the suture line, the occurrence of bone deformities, as well as factors resulting from inflammation, such as fever, anorexia, lethargy, and infections [8].

There are no specific treatments for surgical dehorning wounds in bovines. However, the most frequent treatments in this type of lesion are topical application of healing, antimicrobial, repellent drugs, and still zinc oxide [9], aluminum-based products [10], calcium and sodium alginate-based dressings, iodinated solutions, silver sulfadiazine products, and potassium permanganate-based detergent solutions [11].

A relatively recent biotechnological alternative to wounds treatment is the use of biomaterials with different mechanical, physicochemical and biological properties, including tissue regeneration, drug delivery systems, new vascular grafts or supports for tissue engineering in vitro and in vivo [12]. These materials are usually composed of biopolymers and they can be used as an alternative for wound and burn dressings. Biomaterials exhibit high biocompatibility and biodegradability, as well as similarity to extracellular matrix [13].

According to Rambo et al. [14], bacterial nanocellulose (BNC) is a biopolymer of nanofibrillar structure with similarity to extracellular matrix. Ataide et al. [15] stated that BNC has the desirable properties for wound healing, such as high purity and water-holding capacity, as well as being moldable. Klemm et al. [16] and Trovatti et al. [17] complemented by including high mechanical strength and three-dimensional porosity.

On the other hand, BNC membrane alone does not possess anti-inflammatory or antimicrobial activity [18]. However, some works have been effective when bioactive substances or molecules are incorporated into BNC [19]. Chitosan-associated BNC membranes showed significant inhibition of *Escherichia coli* and *Staphylococcus aureus*, as well as a stimulus to epithelialization and regeneration of surgical wounds in rats [20]. Moreover, the use of bacteriocins has been an alternative to combat etiological agents resistant to the most widely used antimicrobials [21,22]. Among the bacteriocins, nisin is particularly noteworthy [23]. Some studies demonstrate therapeutic potential of its applications [21,24] in injuries in humans [25], and in the Veterinary Medicine [26,27,28].

Therefore, this study aimed to unprecedentedly evaluate the efficiency of using two new complementary treatments—BNC membranes and BNC membranes loaded with nisin—for wound healing in surgical dehorning in bovines, as well as the safety of the biomaterials, considering the biocompatibility of the membrane. The great challenge of this work was to bring, for the first time, BNC membranes—an extremely versatile biomaterial—to be applied in bovines as a dressing matrix and as a drug release for nisin.

## 2. Materials and Methods

### 2.1. Preparation of BNC Membranes and Incorporation of Nisin

The BNC membranes were produced by cultivating the bacterium *Gluconacetobacter xylinus* ATCC 53582 in Hestrin and Schramm medium (20 g/L glucose, 5 g/L bacterial peptone, 5 g/L yeast extract, 2.7 g/L anhydrous sodium phosphate, 1.5 g/L citric acid monohydrate). The culture was performed in a 250 mL Erlenmeyer flask. The inoculated volume was 10 mL of medium containing approximately 107 CFU (Colony Forming Units) of *Gluconacetobacter xylinus*. The flasks were kept for 7 days in static culture at 30 °C. Membranes approximately 2 mm thick were obtained.

After growth, the membranes were immersed in 1M sodium hydroxide (NaOH) solution under stirring at 60 °C for 2 h. Subsequently, they were washed in distilled water. The membranes were then autoclaved at 121 °C for 15 min in distilled water and 1% phosphate buffer solution (PBS) and stored at 4 °C, according to the protocol proposed by Jozala et al. [29].

Nisin from *Lactococcus lactis* at 2.5% purity was purchased commercially (Sigma-Aldrich, St. Louis, MO, USA), with 10^6^ AU activity (AU refers to arbitrary units of activity defined per inhibition halo). The standard solution of nisin 25 µg/mL was prepared in 1 L of sterile PBS at pH 7. Each membrane was previously sterilized and then immersed in 10 mL of the 25 µg/mL nisin solution in an aseptic environment. The flasks were stirred continuously at 100 rpm for 4 h at 25 °C. After this period, the membranes were removed and kept at 4 °C according to a protocol adapted from Dos Santos et al. [30].

### 2.2. Study Design and Characterization of the Animals

This is an experimental study with bovine animals in which the surgical dehorning procedure was idealized as a model for evaluation and comparison of the macroscopic and microscopic behavior of surgical wounds regarding the inflammatory phase of the healing process. The project was submitted to the Ethics Committee on Animal Use of the University of Sorocaba—CEUA/UNISO, Sorocaba, São Paulo State, Brazil, and approved under protocols numbers 162/2019 and 149/2019.

For this study we used 24 cows having Nelore characteristics and crossbreds from the farm Estância Hortência, located in the city of Salto de Pirapora, São Paulo State, Brazil. These animals were from two to ten years old and had lived for at least 12 months in the farm; hence, they were properly adapted to the production and handling system.

The animals were kept from 60 days pre-surgery to 60 days post-surgery in an intensive grazing system. In this type of system, cows are periodically moved from one picket to another according to forage consumption, in a rotation system. This method ensures excellent feeding and animal welfare conditions for the cows, since it prioritizes the use of technologies for efficient and productive management. In addition, during the whole time they are on the pickets, the animals receive water ad libitum and mineral supplementation.

The animals were selected by Body Condition Score (BCS), negative pregnancy diagnosis, and by the previous assessment of the surgeon responsible for the dehorning procedure based on the horn diameter in relation to the horn base, in order to ensure the dermorrhaphy would be performed minimizing tension at the suture line.

The study was divided into two experiments: I and II. For experiment I, 12 animals were used. The dermorrhaphy-related surgical wounds of 12 left-sided horns received a standard treatment, consisting in this study of cleaning with gauze and antisepsis with topical iodinated polyvinylpyrrolidone (PVPI) solution followed by larvicidal repellent every 48 h. This group was named as Control. The dermorrhaphy-related surgical wounds of 12 right-sided horns received the application of BNC membrane at the base of the cornual process of the frontal bone followed by standard treatment.

For experiment II, another 12 animals were used; the surgical wounds of 12 left side horns received BNC at the base of the cornual process of the frontal bone; the dermorrhaphy related to the surgical wounds of 12 right side horns received BNC+nisin membrane. The standard treatment was also applied to both horns.

### 2.3. Surgical Procedure and Post-Surgery

The surgery included dehorning and dermorrhaphy procedures, and it was conducted individually. Surgery was performed by a single experienced professional trained in the proposed technique. Each animal was previously restrained on a trunk and submitted to sedation with 2% xylazine hydrochloride at a dose of up to 0.2 mg/kg via intramuscular (IM).

For the surgery, the animal was brought down with ropes using the Almeida Barros technique, and kept in a sternal decubitus position with the head flexed to the left for the procedure. Local infiltrative analgesia was achieved with 2% lidocaine hydrochloride without vasoconstrictor for sensory block of the temporal zygomatic branch of the cornual nerve, in addition to the subcutaneous application of 30 mL of the same local anesthetic in the caudal region at the base of each horn.

The surgical procedure was based on the technique proposed by Cunha et al. [7]. This technique consists in the formation of two half-ellipses at the base of the horn and subsequent release through divulsion throughout the horn base between skin and subcutaneous tissue. The horn base is then sawed off and the horn and the half-ellipses are removed, facilitating the dermorrhaphy by reducing the tension of the skin suture. For the dermorrhaphy, a 0.60 nylon thread was used with a Sultan suture pattern (X suture).

The immediate post-operative treatment protocol was performed with a single dose of anti-tetanus serum, intramuscular (IM) administration of the non-steroidal anti-inflammatory drug flunixin meglumine 2.2 mg/kg, as well as antibiotic therapy with benzathine penicillin at a dose of 40,000 IU/animal. Following the postoperative treatment, all the animals were treated with non-steroidal anti-inflammatory drug diclofenac sodium at 5 mg/kg, once a day, for two consecutive days, in addition to benzathine penicillin at 40,000 IU/animal, with an application interval of 48 h, totaling two more applications. These therapeutic protocols are fundamental aiming the animal welfare.

The dynamics standardized treatment for all wounds occurred by cleaning with gauze and antisepsis with a topical PVPI solution followed by larvicidal repellent every 48 h or upon evaluation by the surgeon in charge, with the animal contained in a restraining trunk. The surgical stitches were removed only 15 days after performing the biopsies.

### 2.4. Macroscopic Evaluation

For macroscopic analysis, a protocol was established for recording images of the lesions every 48 h from the surgical procedure until the biopsies. Pictures were taken with animals properly restrained in a trunk. After contention of the animals, the wounds were antiseptically cleaned with topical PVPI solution and distilled water, and subsequently dried with gauze.

To standardize image acquisition, a rectangular card (30 × 15 cm) made of millimeter-sized paper with a central cutout (15 × 7 cm) was developed to position the wound. The card was positioned so that the wound remained in the cutout area. The images were then captured using a smartphone camera (Samsung Galaxy Note10 Plus) at approximately 15 cm from the wounds, parallel to the card. Based on this protocol, a dynamic macroscopic comparison was established among the wounds on days 2, 6, 10, and 14 after the surgical procedure, excluding from this evaluation the animals submitted to biopsies on days 3 and 7 after the surgeries.

The 48 images were compiled and organized in a power point file for comparative assessment of the surgical wounds. Based on the obtained photos the team researchers developed a wound condition classification score, with grades ranging from 1 to 4, being (1) moderate inflammation without epithelialization; (2) mild inflammation, initial epithelialization; (3) absence of inflammation, presence of epithelialization; and (4) hair growth, evidence of epithelialization. Thus, a semiquantitative analysis of the slides was performed using the scores.

All 48 images were randomly arranged, and a PowerPoint file with images was sent to six blinded evaluators: three veterinarians (professors from University of Sorocaba not involved with the project) and three students of Veterinary Medicine (students from University of Sorocaba, enrolled in the 9th semester of the course, not involved with the project). Evaluators should considerate the local inflammation process and the condition of the wound edge healing process. For alignment, prior to the assessment the photos with standardized scores were sent to the evaluators.

Clinical evaluation of bovines was conducted concomitantly to the recording of the images; every 48 h the wounds were evaluated regarding the presence of alterations that could indicate toxicity or rejection to the new treatments proposed, such as exuberant inflammatory reactions, mucopurulent infectious processes, abscesses, or even expulsion of the BNC. Furthermore, procedures to check suture quality, sinus percussion, tracheal auscultation, Body Condition Score (BCS) assessment, and weighing of the animals were performed at the same intervals.

### 2.5. Microscopic Evaluation

On days 03, 07, 14, and 21 after the surgical procedures in both experiments (I and II), biopsies were carried out using punches. In this study, 8 mm diameter punches were used, and the biopsies were performed by the veterinarian. The tissue samples obtained from the punches were used for microscopic analysis of the lesions. On each established day, a sample number of 3 (three) animals from each experiment was submitted to biopsy, without repeating the procedure on the same animal (since biopsy per se induces injury and a new healing process). Thus, interference in the microscopic analysis by a possible inflammatory process originating from the punch was avoided. After the biopsy, the tissue samples were placed in buffered formalin and sent for histological analysis.

Samples were kept in 10% buffered formalin for 24 h for histological analysis. At the end of this time, they were placed in a water container overnight and, after this step, the samples were left in ethanol 70% until processing. After the fixation stage, the tissue processing was performed by the Automatic Tissue Processor and the specimens were embedded in histological paraffin.

The histological cuts of the wound punches were made in standard microtome with 4 µm thickness. Thereafter, the slides were stained by hematoxylin and eosin technique and mounted in histological resin [31]. The slides were evaluated under a light microscope by a pathologist (single-blinded condition). A semiquantitative analysis of the slides was performed using scores of Inflammation Grades (IG): IG 1 (mild inflammation), IG 2 (moderate inflammation), and IG 3 (severe inflammation).

### 2.6. Statistical Analysis

Since data are reported as scores, the results were presented as percentage (%) of animals in each score. When the groups or even the healing times were compared, Wilcoxon’s test or Chi-square test were used and *p*-values < 0.05 were considered statistically significant. The results were analyzed using the Stata^®^ software (version 11.2, College Station, TX, USA).

## 3. Results

**Figure 1:**
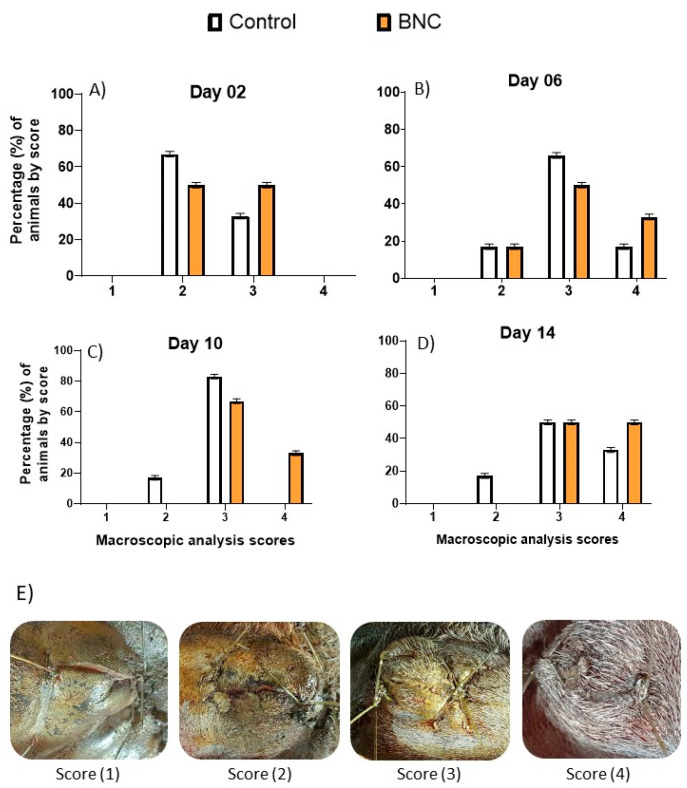
Descriptive macroscopic evaluation of the surgical wounds of animals from Experiment I (*n* = 12) by score at 2 days (**A**), 6 days (**B**), 10 days (**C**), and 14 days (**D**) post-surgery. In letter (**E**), typical images for each score, been: 1—Moderate inflammation, no epithelialization; 2—Mild inflammation, initial epithelialization; 3—No inflammation, presence of epithelialization; 4—Hair growth, signs of epithelialization. Control group = standard treatment (antisepsis with topical PVPI solution followed by larvicidal repellent); BNC group = standard treatment + bacterial nanocellulose membrane.

Four different post-surgical times were evaluated for the macroscopic follow-up of wounds in both experiments. The wound healing evolution of the animals belonging to Experiment I is reported in Figure 1.

A descriptive analysis showed that on day 2 (Figure 1A) a higher percentage of animals in the control group (67%) were in score 2, whereas 50% of the animals receiving the BNC were in score 3. On day 6 (Figure 1B), 33% of the animals in the BNC group had a healing score of 4, compared to 17% of the control group. On day 10 (Figure 1C), there was a decrease in the healing process in Control, as 83% of the animals were within score 3, compared to 33% of BNC in score 4. Finally, on day 14 (Figure 1D), 33% of the animals in the Control group showed hair growth and signs of epithelialization (score 4) compared to 50% of the animals in BNC.

**Figure 2:**
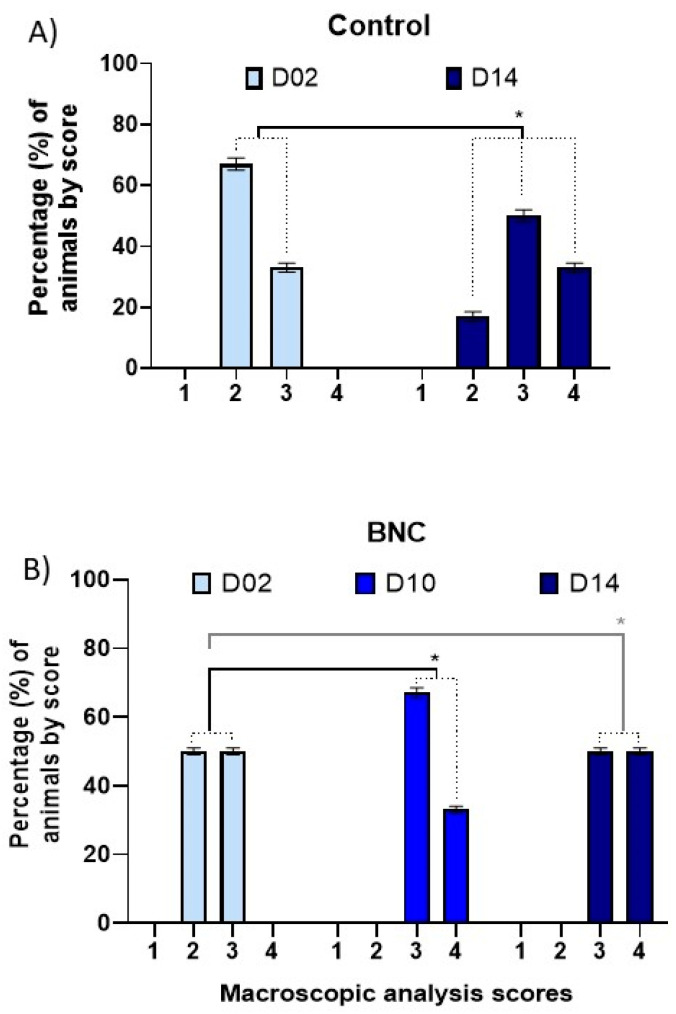
Influence of time for intragroup analysis of healing process (*n* = 12). Macroscopic evaluation of surgical wounds of animals from Experiment I. In (**A**) Control group = standard treatment (antisepsis with topical PVPI solution followed by larvicidal repellent); comparison of data between days 2 and 14; * *p* < 0.05. In (**B**) BNC group = standard treatment + bacterial nanocellulose membrane; comparison of data among days 2, 10 and 14; * *p* < 0.05.

Concerning statistical analysis, the influence of time on the healing process was first evaluated without comparing treatments. Figure 2 summarizes the differences between day 2 and 14 for control, and among day 2, 10, and 14 for the BNC group. For this purpose, the horns of the control group were assessed over the times 6, 10, and 14 days in relation to day 02 (the same horns over time). There was no significant difference in the healing process between days 2 and 6. Comparing days 2 and 10, no significant difference in healing was observed either, regardless of *p*-value = 0.0833. However, when data were compared between days 2 and 14, a statistically significant difference (*p* = 0.0495) was evidenced between the final and initial healing (Figure 2A).

On the other hand, evaluating only the horns of the BNC group over the days, it was observed that between days 2 and 6 there was no significant difference in the healing process. However, when comparing days 2 and 10, a significant difference (*p* = 0.0495) was noted between the heals, thus evidencing the acceleration of the healing process using membrane, while in control this healing only occurred on day 14. The analysis comparing day 2 and 14 also showed a significant difference (*p* = 0.0495) between the final and initial healing.

Finally, a paired evaluation between the control and the BNC horns was performed. On day 2, no significant difference was observed, as well as in the evaluation conducted on day 6. Analyses on days 10 and 14 also did not reveal statistical difference regarding healing, that means the use or not of BNC does not improve the healing process considered paired evaluation. However, when the horns of each group were their own control, the horns with BNC healed faster.

**Figure 3:**
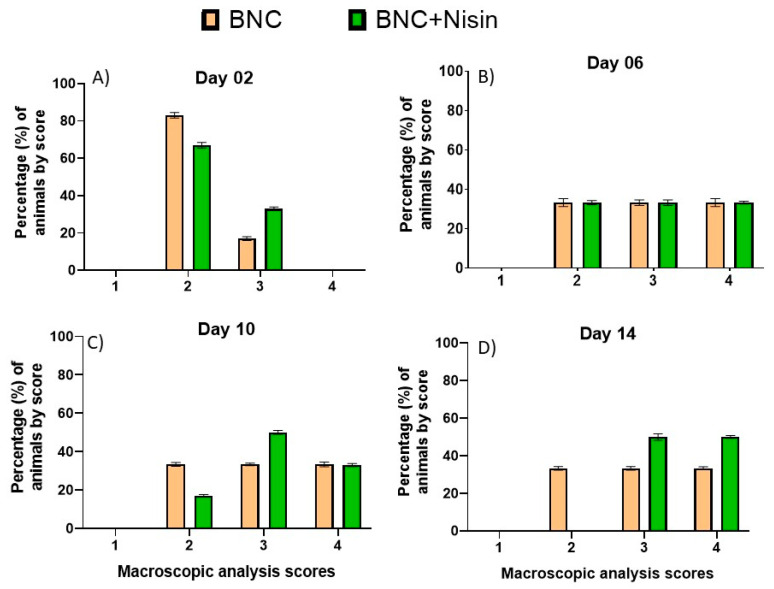
Descriptive macroscopic evaluation of the surgical wounds of animals from Experiment II (*n* = 12), by score, at 2 days (**A**), 6 days (**B**), 10 days (**C**) and 14 days (**D**) post-surgery. Scores: 1—Moderate inflammation, no epithelialization; 2—Mild inflammation, initial epithelialization; 3—No inflammation, presence of epithelialization; 4—Hair growth, signs of epithelialization. BNC group = standard treatment + bacterial nanocellulose membrane; BNC+Nisin = standard treatment + Nisin loaded in bacterial nanocellulose membrane.

Regarding Experiment II (BNC and BNC+nisin), the evolution of healing is reported in Figure 3. In day 2 (Figure 3A) 33% of the animals from the BNC+nisin group had a healing score of three compared to 17% of the animals in the BNC group. On day 6 (Figure 3B) both groups exhibited the same percentages of animals/score. On day 10 (Figure 3C), the same percentage of animals were at a healing score of four, while on day 14 (Figure 3D), 50% of the animals in the BNC+nisin group were at score four, as compared to 33% of the BNC group.

**Figure 4:**
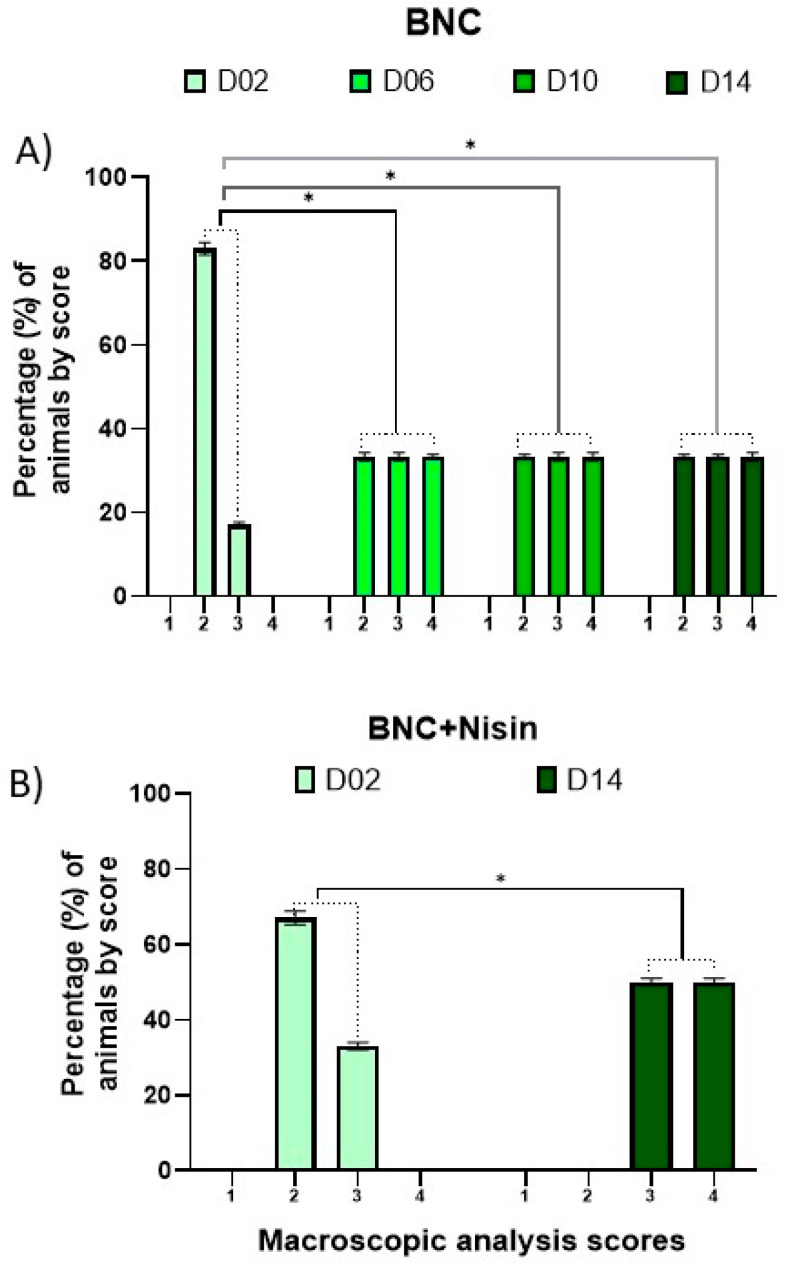
Influence of time for intragroup analysis of healing process (*n* = 12). Macroscopic evaluation of surgical wounds of animals from Experiment II. In (**A**) BNC group = standard treatment + bacterial nanocellulose membrane; comparison of data among days 2, 6, 10 and 14; * *p* < 0.05. In (**B**) BNC+Nisin group = standard treatment + Nisin loaded in bacterial nanocellulose membrane; comparison of data among days 2 and 14; * *p* < 0.05.

Statistical analyses of time and comparison between groups were also performed for Experiment II. The influence of time on the healing process was evaluated with the horns of the same group over time 6, 10, and 14 days in relation to day 2 (Figure 4). A significant difference was observed in the membrane group from day 6 compared to day 2 (*p* = 0.0495). The statistical difference was maintained between days 2 and 10 (*p* = 0.0495) and days 2 and 14 (*p* = 0.0495), i.e., there was an improvement in the healing process as early as day 6 of the evaluation.

Regarding the BNC+nisin group, no statistical difference was observed in the healing process between days 2 and 6. Moreover, between days 2 and 10, there was no significant difference between the heals. In contrast, between days 2 and 14, a significant improvement (*p* = 0.0495) was observed. This finding suggests that nisin loaded in BNC was not able to improve healing time, as BNC alone presented faster healing results. Figure 4 depicts the data of statistical differences from day 2 in the BNC group and between days 2 and 14 in the BNC+nisin group.

Paired evaluation between the BNC and BNC+nisin horns showed that on day 2 there was no significant difference between the groups, as well as in the evaluation performed on days 6, 10, and 14 (all *p* > 0.05). These results indicated that regardless of the time both treatments had similar paired evolution.

Wounds were microscopically evaluated at three different moments in both experiments (it was not possible to process the samples from the third day), and they were evaluated according to inflammation grades (IG). A descriptive analysis reveals that on day 7, 67% of the animals in the control group were represented by moderate IG, which corresponded to the same percentage of the animals that received the BNC membrane application. On day 14, 100% of the animals in the control group had moderate IG compared to 67% of the BNC group on this day. On day 21, there was an evolution in the healing process in the control group, as 67% of the animals had mild IG, when compared to 33% of the animals having BNC in the same degree of inflammation. Nonetheless of the group, it could be observed that up to 21 days the inflammation, although mild in some cases, inflammation was still markedly present.

The influence of time on the histologic healing process was assessed with the horns of the same group over time as compared to day 7. For the control group, there was no significant improvement on day 14 or 21 when compared to initial day (*p* = 0.3173 and 0.1655, respectively). Similarly, in the BNC group there was no significant difference in the histological analysis on days 14 and 21 (*p* = 1.0 and 0.7815, respectively) in relation to day 7.

Concerning the paired analyses between groups, no statistical difference was found in any of the times evaluated. It was noticed that these collection times were not sufficient for an improvement in the initial inflammatory process, and consequently to evaluate wound healing.

**Figure 5:**
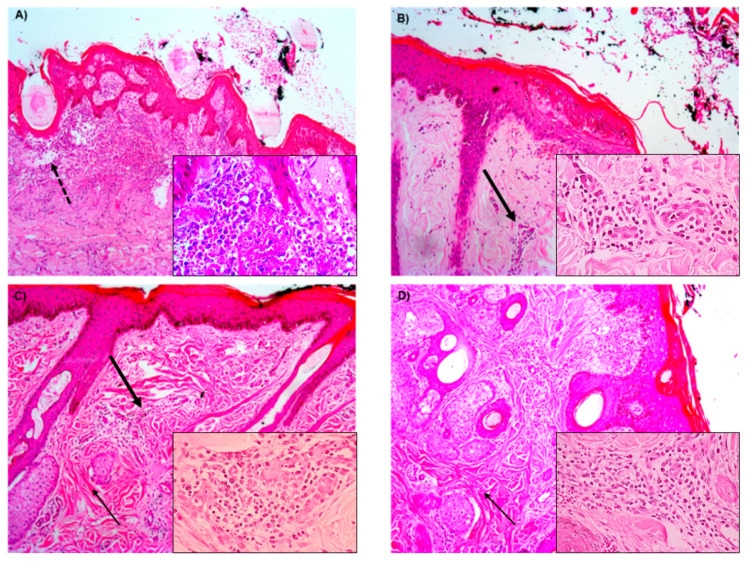
Photomicrographs of the slides at the different times of the skin biopsies stained by hematoxylin and eosin at 100× and 400× (insets) magnification; (**A**) animal in the control group at 7 days, with active inflammation, characterized by marked inflammatory infiltrate (inset); (**B**) animal in the membrane group, 7 days, with chronic inflammation, noting the beginning of tissue reorganization and decrease of inflammatory cells with mononuclear pattern (inset); (**C**) animal in the membrane group, 14 days, with chronic-active inflammation (inset), with areas of fibrosis and edema; (**D**) animal in the membrane+nisin group, 21 days, with chronic-active inflammation (inset), greater tissue reorganization, areas of fibrosis, and cellular accumulation of restructuring. Note: the dotted arrow indicates area of edema; thick arrow indicates area of inflammatory infiltrate; thin arrow indicates area of fibrosis.

The photomicrographs of the slides (Figure 5) mostly presented inflammation ranging from active to chronic-active, showing discrete, moderate, or intense inflammatory infiltrates of mononuclear, polymorphonuclear, or mixed cells. The areas of edema or congestion of the superficial dermis described for the samples on day 7 decreased according to the days of biopsy, as expected. The slides related to the punches taken on day 21 post-procedure revealed predominantly areas of calcification and fibrosis in the deep dermis associated with mononuclear inflammatory infiltrates. The evaluation of the slides took into consideration the inflammatory characteristics described above, as well as the inflammation grades (IG 1, IG 2, and IG 3).

Descriptive healing evolution by microscopic analysis, in Experiment II, showed in day 7 100% of animals in BNC group reaching moderate IG, compared to 67% of animals in the BNC+nisin group. On day 14, there was regression of the inflammatory process in BNC, with 67% of animals in moderate IG and 33% in severe GI. On day 21, the BNC group returned to 100% of animals in moderate IG, and 33% of animals in BNC+nisin developed into a mild IG response.

The influence of time on the histological healing process was evaluated with the horns of the same group over time compared to day 7. There was no statistically significant difference in terms of the inflammation grade at any of the evaluated times (14 and 21 days) in comparison to day 7, both in the BNC group and in the BNC+nisin group. Likewise, no significant difference was found in the paired analyses between groups.

It was observed that these collection times were not enough to evaluate the healing process. Slides showed chronic-active inflammatory characteristics in both groups, with cases of inflammation associated with neutrophilia in most slides, which showed mild, moderate, or intense inflammatory infiltrates of mononuclear, polymorphonuclear, or mixed cells with areas of calcification and neovascularization, and multifocal fibrosis.

## 4. Discussion

BNC is a highly hydrated hydrogel. Its fibrous structure consists of a three-dimensional network of nanofibers. This nanometric morphology provides bacterial cellulose with a large surface area, high liquid retention and absorption capacity, elasticity, and malleability [16,32]. Moreover, this biopolymer is biodegradable, non-toxic and non-allergenic [33], with a high degree of biocompatibility [34].

Due to these previously mentioned properties, BNC appears as an ideal matrix for use in many fields related to biomedical and biotechnological applications, and, for the first time, in large animals, such as bovines. In the present study, the use of the BNC membrane accelerated the macroscopic healing process when the wounds were compared in the same group over time. BNC can create a barrier between the wound and the environment, because of its cross-linked and porous structure, thereby preventing bacterial infections [35].

Despite the microscopic data showing no difference between groups or even over the 21 days of analysis, Fu, Zhang, and Yang [32] suggested the membrane has a high capacity to absorb exudate during the inflammatory phase of healing, and therefore has great potential for application in wound healing. In study involving rats, faster epithelialization of surgical wounds treated with BNC were obtained relative to the control group, and a complete closure of the lesion around 15 days after application [20].

Park et al. [36] compared the effect of the application of BNC membranes, vaseline, and alginate dressings in rats through surgical skin excisions and application in dorsal region; they observed a decrease in the inflammatory phase of healing using the membrane by the lower neutrophil infiltrate and higher fibroblastic activity for wound contraction, thus accelerating the healing process. Our data allow us to suggest that the application of the BNC in experiment I revealed, macroscopically, acceleration of healing when compared to the control horns, since at 10 days after the procedure, in the Membrane group, the wounds were visually healed, whereas in the control group healing only occurred after evaluation at 14 days.

The same relation cannot be established for experiment II, nor in the microscopic evaluation. Some factors may be taken into consideration for our study to have revealed similar data in the macroscopic evaluation to those presented by the mentioned authors, but without corroborating the results obtained in the microscopic analysis. Surgical dehorning was used as an experimental model of surgical wound; the choice of this procedure occurred due to the need to perform the procedures to improve animal handling and reduce traumatic disputes in troughs, which caused injuries and decreased production in the animals of the farm. In addition, carrying out the horn removal on the animals allowed us to perform a comparison in the same animal using different treatments (control, BNC, and BNC+nisin).

According to Lin et al. [20] and Park et al. [36] the dressings were applied after surgical skin excisions and periodically changed during the evaluations, a fact that did not occur in our study, since the application of the membranes occurred during the surgical procedures, with their placement at the base of the cornual process of the frontal bone. This way of evaluating the action of the membranes was also unprecedented, without replacement throughout the recovery of the animal.

Wouk et al. [37] evaluated the action of different therapeutic agents for healing in pigs by producing experimental skin lesions by performing biopsies on given days. These procedures occurred in a controlled environment, suggesting that these differences, especially regarding the microscopic aspect, may be related to the invasive characteristic of the dehorning surgery, which establishes a much greater challenge for the membrane when compared to its application with topical dressings or in skin excisions, using procedures for manipulation of the surgical wound to remove the horns and divulsion of the skin in order to ensure adequate tension of the suture line. 

These factors may have contributed to the difficulty in activating the potential of acceleration of the inflammatory phase of wound healing by BNC, as well as possible contamination, which is relatively frequent in surgeries performed in the field. Another important point is related to the fact that the animals, as soon as they recovered from the surgeries, returned to the paddocks for grazing, to maintain the natural handling characteristic of the animals.

In experiment II, the natural antimicrobial nisin, produced by bacteria such as *Lactococcus lactis*, was loaded in BNC [38]. The therapeutic potential of nisin has been widely explored [21,24] both in humans [25] and for veterinary medicine, with nisin activity on agents such as methicillin-resistant *Staphylococcus aureus* (MRSA) and vancomycin-resistant *Enterococcus* (VRE) [26,27,28].

The release of this bacteriocin into bacterial nanocellulose membranes for acceleration of the skin wound healing process in rats was evaluated by Heunis, Smith, and Dicks [39]. The authors observed a significant difference in wound closure in topical nisin loaded in polymer skin dressings when compared to the control with gauze dressings between days 4 and 7 of surgical excision. Nisin and other bacteriocins were reported as effective in treating skin lesions caused by *Staphylococcus aureus*, *Propionibacterium acnes*, *Staphylococcus epidermidis*, *Bacillus cereus*, *Bacillus subtilis*, and *Listeria monocytogenes* [40]. According to Mastromatteo et al. [41], the activity of nisin against Gram-negative bacteria occurs satisfactorily when associated with chelating agents.

Although the statistical difference in nisin application was not observe on the dermorrhaphy model, bacteriocin still has uses capability, especially for not causing dysbiosis. Nisin utilization can affect the composition of microbiota and its metabolic activities, promoting host health and/or prevent diseases. This finding was reported by Francino et al. [42], who demonstrated bacteriocins can balance the microbiota with healthy bacterial populations, and they prevent dysbiosis generated by broad-spectrum antibiotics. Moreover, according to Radaic and colleagues [43], nisin enhanced healthier oral microbiome and decreased the levels of pathogens.

The antioxidant activity of nisin was studied by Dos Santos et al. [30] who suggested its use associated with biopolymers such as BNC membrane, as they act as a platform for gradual release of nisin and microbial growth control. In the study, the authors performed immersion of BNC membranes in concentrations ranging from 15 to 550 µg/mL of nisin with and without ethylene diamine tetra-acetic acid (EDTA) and observed antimicrobial activity (restricted to *Staphylococcus aureus*) only at concentrations starting at 63 µg/mL without EDTA. In our study, no significant difference was found when comparing the use of membranes with and without nisin in dehorning surgical wounds. The concentration used was 25 µg/mL, a fact that corroborates the results found by the cited authors.

## 5. Conclusions

For the first time, it was performed the application of BNC as a dressing matrix and as a drug release for nisin in surgical dehorning wounds in bovines, followed by evaluating the efficacy of the BNC treatment in a real breeding environment. The most of studies with BNC are associated with small species (usually guinea pigs) maintained in experimentation vivarium, that means in laboratorial environment.

There was a clinical improvement in the healing response with the use of BNC when compared to the control. However, there was no significant difference between the use of the BNC compared to the BNC+nisin. Histologically, there was also no significant difference between the groups studied. The time of evaluation was short, and the microscopic healing did not follow the one observed macroscopically. Further studies are needed to evaluate the use of antibiotic loaded in BNC membranes in the healing process of bovine skin, using other doses of nisin and employing a longer time of analysis.

## Data Availability

The data presented in this study are available on request from the corresponding author.

## References

[1] World Organisation for Animal Health (OIE) (2019). Animal welfare and beef cattle Production Systems. Terr. Anim. Health Code.

[2] Devries T., Von Keyserlingk M., Beauchemin K. (2005). Frequency of Feed Delivery Affects the Behavior of Lactating Dairy Cows. J. Dairy Sci..

[3] Huzzey J., Von Keyserlingk M., Weary D. (2005). Changes in Feeding, Drinking, and Standing Behavior of Dairy Cows During the Transition Period. J. Dairy Sci..

[4] Stafford K., Mellor D. (2005). Dehorning and disbudding distress and its alleviation in calves. Vet. J..

[5] Weaver A.D., St. Jean G., Steiner A. (2005). Head and Neck Surgery. Bovine Surgery and Lameness.

[6] Sylvester S.P., Stafford K.J., Mellor D.J., Bruce R.A., Ward R.N. (1998). Acute cortisol responses of calves to four methods of de-horning by amputation. Aust. Vet. J..

[7] da Cunha O., da Cunha A.F., de Souza C., Cousseau I., Galli M.A., Rodaski S., Svoboda W.K., dos Sntos R. (2002). Nova técnica para descorna em bovinos. Arquivos De Ciências Veterinárias E Zoologia Da UNIPAR.

[8] Da Silva L.A.F., Teixeira Neto A.R., Campos S.B.S., Brazil D.S., Helou J.B., Pucci R.L., Caetano L.B., Maranhão R.P.A., Brandstetter L.R.G. (2009). Epidemiological aspects of sinusitis after plastic or burning iron dehorning in cattle. Acta Sci. Vet..

[9] Cangul I.T., Gul N.Y., Topal A., Yilmaz R. (2006). Evaluation of the effects of topical tripeptide-copper complex and zinc oxide on open-wound healing in rabbits. Vet. Dermatol..

[10] Rahmati M., Mozafari M. (2019). Biocompatibility of alumina-based biomaterials-A review. J. Cell. Physiol..

[11] Brawn K. (2015). Guidelines for the Assessment & Management of Wounds.

[12] Tottoli E.M., Dorati R., Genta I., Chiesa E., Pisani S., Conti B. (2020). Skin Wound Healing Process and New Emerging Technolo-gies for Skin Wound Care and Regeneration. Pharmaceutics.

[13] Balakrishnan B., Jayakrishnan A. (2005). Self-cross-linking biopolymers as injectable in situ forming biodegradable scaffolds. Biomaterials.

[14] Rambo C., Recouvreux D., Carminatti C., Pitlovanciv A., Antonio R., Porto L. (2008). Template assisted synthesis of porous nanofibrous cellulose membranes for tissue engineering. Mater. Sci. Eng. C.

[15] Ataide J.A., De Carvalho N.M., Rebelo M.D.A., Chaud M.V., Grotto D., Gerenutti M., Rai M., Mazzola P.G., Jozala A.F. (2017). Bacterial Nanocellulose Loaded with Bromelain: Assessment of Antimicrobial, Antioxidant and Physical-Chemical Properties. Sci. Rep..

[16] Klemm D., Kramer F., Moritz S., Lindström T., Ankerfors M., Gray D., Dorris A. (2011). Nanocelluloses: A new family of na-ture-based materials. Angew. Chem. Int. Ed..

[17] Trovatti E., Serafim L.S., Freire C.S., Silvestre A.J., Neto C.P. (2011). Gluconacetobacter sacchari: An efficient bacterial cellulose cell-factory. Carbohydr. Polym..

[18] Maneerung T., Tokura S., Rujiravanit R. (2008). Impregnation of silver nanoparticles into bacterial cellulose for antimicrobial wound dressing. Carbohydr. Polym..

[19] Jebel F.S., Almasi H. (2016). Morphological, physical, antimicrobial and release properties of ZnO nanoparticles-loaded bacterial cellulose films. Carbohydr. Polym..

[20] Lin W.-C., Lien C.-C., Yeh H.-J., Yu C.-M., Hsu S.-H. (2013). Bacterial cellulose and bacterial cellulose–chitosan membranes for wound dressing applications. Carbohydr. Polym..

[21] Allen H.K., Trachsel J., Looft T., Casey T.A. (2014). Finding alternatives to antibiotics. Ann. N. Y. Acad. Sci..

[22] Rolain J.-M., Abat C., Jimeno M.-T., Fournier P.-E., Raoult D. (2016). Do we need new antibiotics?. Clin. Microbiol. Infect..

[23] Gharsallaoui A., Joly C., Oulahal N., Degraeve P. (2013). Nisin as a Food Preservative: Part 2: Antimicrobial Polymer Materials Containing Nisin. Crit. Rev. Food Sci. Nutr..

[24] Behrens H.M., Six A., Walker D., Kleanthous C. (2017). The therapeutic potential of bacteriocins as protein antibiotics. Emerg. Top. Life Sci..

[25] Van Heel A.J., Montalban-Lopez M., Kuipers O.P. (2011). Evaluating the feasibility of lantibiotics as an alternative therapy against bacterial infections in humans. Expert Opin. Drug Metab. Toxicol..

[26] Cintas L.M., Casaus M.P., Herranz C., Nes I.F., Hernández P.E. (2001). Review: Bacteriocins of Lactic Acid Bacteria. Food Sci. Technol. Int..

[27] Field D., Seisling N., Cotter P.D., Ross R.P., Hill C. (2016). Synergistic nisin-polymyxin combinations for the control of pseudo-monas biofilm formation. Front. Microbiol..

[28] Piper C., Cotter P., Ross R., Hill C. (2009). Discovery of medically significant lantibiotics. Curr. Drug Discov. Technol..

[29] Jozala A.F., Pértile R.A., dos Santos C.A., de Carvalho Santos-Ebinuma V., Seckler M.M., Gama F.M., Pessoa A. (2015). Bacterial cellulose production by *Gluconacetobacter xylinus* by employing alternative culture media. Appl. Microbiol. Biotechnol..

[30] Jozala A.F., de Lencastre-Novaes L.C., Lopes A.M., de Carvalho Santos-Ebinuma V., Mazzola P.G., Pessoa A., Grotto D., Gerenutti M., Chaud M.V. (2016). Bacterial nanocellulose production and application: A 10-year overview. Appl. Microbiol. Biotechnol..

[31] Tolosa E.M.C., Rodrigues C.J., Behmer O.A., Freitas Neto A.G. (2003). Manual de Técnicas Para Histologia Normal e Patológica.

[32] Fu L., Zhang J., Yang G. (2013). Present status and applications of bacterial cellulose-based materials for skin tissue repair. Carbohydr. Polym..

[33] Klemm D., Heublein B., Fink H.-P., Bohn A. (2005). Cellulose: Fascinating Biopolymer and Sustainable Raw Material. Angew. Chem. Int. Ed..

[34] Sanchavanakit N., Sangrungraungroj W., Kaomongkolgit R., Banaprasert T., Pavasant P., Phisalaphong M. (2006). Growth of Human Keratinocytes and Fibroblasts on Bacterial Cellulose Film. Biotechnol. Prog..

[35] Sulaeva I., Henniges U., Rosenau T., Potthast A. (2015). Bacterial cellulose as a material for wound treatment: Properties and modifications. A review. Biotechnol. Adv..

[36] Park S.U., Lee B.K., Kim M.S., Park K.K., Sung W.J., Kim H.Y., Gil Han D., Shim J.S., Lee Y.J., Kim S.H. (2012). The possibility of microbial cellulose for dressing and scaffold materials. Int. Wound J..

[37] De Figueiredo Wouk A.F.P., Diniz J.M., Círio S.M., Santos H., Baltazar E.L., Acco A. (1998). Membrana biológica (biofill)—estudo comparativo com outros agentes promotores da cicatrização da pele em suínos: Aspectos clínicos, histopatológicos e morfométricos. Arch. Vet. Sci..

[38] de Arauz L.J., Jozala A.F., Mazzola P.G., Penna T.C.V. (2009). Nisin biotechnological production and application: A review. Trends Food Sci. Technol..

[39] Heunis T.D.J., Smith C., Dicks L.M.T. (2013). Evaluation of a Nisin-Eluting Nanofiber Scaffold to Treat *Staphylococcus aureus*-Induced Skin Infections in Mice. Antimicrob. Agents Chemother..

[40] Bowe W.P., Filip J.C., DiRienzo J.M., Volgina A., Margolis D.J. (2006). Inhibition of propionibacterium acnes by bacteriocin-like inhibitory substances (BLIS) produced by *Streptococcus salivarius*. J. Drugs Dermatol. JDD.

[41] Mastromatteo M., Mastromatteo M., Conte A., Nobile M.A. (2010). Advances in controlled release devices for food packaging applications. Trends Food Sci. Technol..

[42] Francino M.P. (2016). Antibiotics and the human gut microbiome: Dysbioses and accumulation of resistances. Front. Microbiol..

[43] Radaic A., Ye C., Parks B., Gao L., Kuraji R., Malone E., Kamarajan P., Zhan L., Kapila Y.L. (2020). Modulation of pathogenic oral biofilms towards health with nisin probiotic. J. Oral Microbiol..

